# Unusual case of lower back pain-piriformis myositis: a case report and literature review

**DOI:** 10.11604/pamj.2019.32.4.17808

**Published:** 2019-01-03

**Authors:** Ahmed Elhagar, Ibrahim Kamar, Mohamed Faisal Hassan Elsheikh, Anant Mahapatra, Tarig Fadlallah Altahir Ahmed, Yogesh Acharya, Khalid Khan

**Affiliations:** 1 Trauma and Orthopedics Department, Our Lady of Lourdes Hospital, Drogheda, Republic of Ireland; 2 Avalon University School of Medicine, Willemstad, Curacao, Antilles Netherlands

**Keywords:** Piriformis pyomyositis, low back pain, case study, review literature

## Abstract

We present a case of a 37-year-old male security officer with fever, severe low back pain radiating to left lower leg and diminished mobility for 1 week. His Lumbar spine X-ray was unremarkable, but his inflammatory markers including CRP, ESR and Neutrophils were high. CT scan with contrast showed rim enhancing fluid collection within the left obturator foramen with inflammatory change in the mesorectal fat. Confirmatory MRI scans depicted inflammatory change in the left piriformis muscle and a localized collection without any abnormality in the spine. Urgent CT guided aspiration was performed and the sample sent for microbiological analysis. Intravenous antibiotics commenced and continued for two weeks with complete resolution.

## Introduction

More than half of all the adults experience low back pain (LBP) at some point in their life, being one of the most common cause of scheduled physician visit [1, 2]. Pyomyositis of pelvic muscle is a rare cause of LBP with an increased morbidity and disability. Inflammatory changes in piriformis, more commonly due to fall or infection, can compress sciatic nerve causing sciatica in patients. We present a case with pyomyositis of left piriformis muscle who presented with low back pain and sciatica.

## Patient and observation

**Figure 1:**
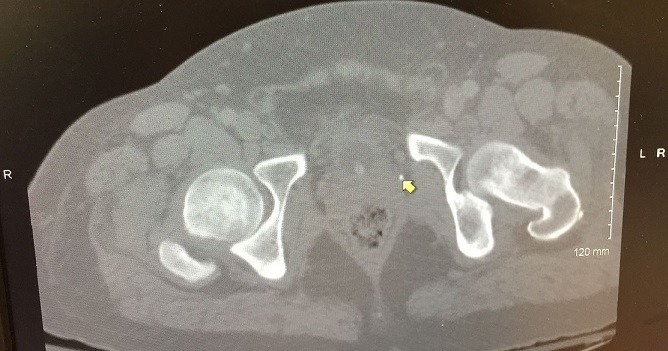
CT-scan abdomen and pelvis (axial section), showing rim enhancing fluid collections in the left obturator foramen approximately 2.5cm in diameter consistent with an abscess (arrow); there is some abnormality in the mesorectal fat along with some stranding extending up from the lower left pelvis along the iliac chain anterior to the psoas

A 37-year-old male Caucasian security officer presented to our emergency department with severe low back pain for seven days radiating to the left lower limb with an associated single episode of urinary retention and constipation. He also complained of difficulty in walking from a day before. On further inquiry, the patient was generally healthy with no significant medical and surgical history. On examination, he was febrile 38.5°c, but no chills and rigors. Straight leg raising test was negative with marked tenderness at the posterior aspect of the left hip joint. Neurological examination revealed mild decrease in muscle power at the left lower limb in L4, L5 and S1 nerve root distribution (Left L4, L5 and S1 power was 4/5). Hematological investigations showed total white blood count (10.7*10^9/l) with 85% neutrophils (9.10*10^9/l) and raised inflammatory markers: erythrocyte sedimentation rate (ESR) (81mm 1^st^ hour) and C reactive protein (CRP) (528mg/l). His electrolytes and lactate levels were within normal limits. The plain radiograph images of his lumbar spine, pelvic and hip x-rays were unremarkable. The CT scan - pelvis with contrast revealed a rim enhancing fluid collection within the left obturator foramen approximately 25 mm in diameter consistent with an abscess and some inflammatory change in the mesorectal fat and stranding extending up from the lower left pelvis along the iliac chain anterior to the psoas (Figure 1). Intravenous antibiotics with Flucloxacillin (2gm IV) and Benzyl penicillin (2.4gm IV) were commenced. Fevers subsided with slow clinical response, and CRP dropped to 418.9mg/l after 48 hours. CT-guided aspiration was planned. While awaiting for CT-guided aspiration for decompression and bacterial samples, confirmatory magnetic resonance imaging (MRI) of the lumbar and sacral region was performed depicting inflammatory change in the left piriformis muscle and a localized collection measuring 20 mm in diameter at the sciatic notch as on CT without any abnormality in the spine. Subsequent CT guided aspiration yielded 3 cc of yellow fluid. *Staphylococcus aureus* was isolated from the aspirate sensitive to the Flucloxacillin. The patient showed a marked clinical improvement after the aspiration and was continued on IV antibiotics for the next two weeks. The blood count and inflammatory markers revert back to normal. He was discharged on oral antibiotics thereafter and called for follow up after two weeks. At the follow up, patient had no further issues without any traces of residual change.

## Discussion

Pyomyositis, also known as tropical pyomyositis, is usually prevalent but not limited to Africa and south pacific [3]. Pyomyositis of piriformis is an uncommon variant of muscular pyomyositis. There is no specific age and sex predominance, and the cases are distributed equally. There are reported cases of piriformis myositis in a swimmer, tennis and rugby player, as a probable consequence of a sport induced trauma [4-6]. Our patient was a security officer and denied any discernible history of recent trauma prior to the onset of symptoms. Although rare, pyomyositis of piriformis in children can present with characteristic “pinpoint pain” [7]. Compressive symptoms owing to inflammatory swelling can lead to piriformis syndrome and sciatica, a shooting down of pain from sacro-lumbar region towards the buttocks. Our patient presented with a severe low back pain for a week which radiated to left lower limb. The diagnosis of pyomyositis is largely based on strong clinical suspicion. Proper clinical diagnosis is challenging as the infection is deep, making palpation or even needle aspiration more difficult. Inflammatory blood markers are helpful but with diagnostic limitations. Very high levels of CRP without definitive clinical findings support non-rheumatic causes like infection or malignancies. Raised inflammatory markers should be investigated aggressively in these patents with different modalities including advanced radiological test to rule out malignancy and potential infections. CRP was raised in our patient but the blood culture was negative.

Ultrasonography can be useful in evaluating suspected abscesses but it may not be able to determine the extent of bony involvement. Plain radiograph of the lumbar spine, pelvic and hip are usually unremarkable, unless there are signs of significant abscess formation, traumatic and degenerative bony changes. Contrast-enhanced computed tomography is useful as the abscess contains a hyperemic peripheral ring which shows enhancement. MRI imaging is useful in defining the soft tissue boundaries and pre-surgical anatomy. It can detect muscle inflammation and abscess with greater sensitivity making it an ideal investigation of choice for diagnosing pyomyositis [8]. We performed CT scan with contrast initially, followed by MRI scan to confirm the diagnosis after slow patient response to antibiotics. Imaging studies revealed pyomyositis of left piriformis muscle and a limited collection at the sciatic notch consistent with an abscess. The plain radiograph images of his lumbar spine, pelvic, and hip were unremarkable in our case.

*Staphylococcus aureus* is the commonest bacteria associated with pyomyositis and it is usually managed with antibiotics. Surgical intervention is needed for drainage of the abscess, worsening symptoms even with antibiotics and an incomplete resolution. Most of the patients will have complete resolution with antibiotics with or without surgical intervention. We performed urgent CT guided aspiration/decompression and *Staphylococcus aureus* was isolated from the aspirate, sensitive to the flucloxacillin. IV antibiotic was commenced and continued for two weeks until complete resolution was achieved. Diagnosis of pyomyositis can be easily missed owing to its varied presentations and overlapping features. It is necessary for physicians and health care practitioners to maintain a high degree of suspicion for its murky clinical course. Diseases like septic arthritis of the hip, epidural abscess and osteomyelitis may mimic pyomyositis posing a diagnostic challenge [9, 10]. Early diagnosis and treatment can prevent the potential complications, such as abscess formation, local extension, bacterial dissemination and neurovascular compression that can lead to disabling conditions like sciatica. Bacteraemia can result in high morbidity and mortality. It is imperative that physicians should have a high degree of vigilance to overcome a diagnostic and therapeutic challenge for attaining a favorable outcome.

## Conclusion

Pyomyositis of piriformis should be considered in a patient with low back pain, sciatica and associated inflammatory symptoms. Although it is a rare infective disease, it attains a complete resolution if treated promptly with intravenous antibiotics. But diagnostic delay can result in deep abscess formation, sepsis and even death. Therefore, it is important for the healthcare workers to have a high diagnostic index for early diagnosis to prevent potential complications and disabling neurovascular compression.

## Competing interests

The authors declare no competing interests.
